# A novel series of conferences tackling the hurdles confronting the translation of novel cancer immunotherapies

**DOI:** 10.1186/1479-5876-10-218

**Published:** 2012-11-05

**Authors:** Adrian Bot, Mark Ahn, Marnix Bosch, Dirk Brockstedt, Lisa H Butterfield, Andrew Cornforth, Richard Harrop, W Martin Kast, Richard Koya, Francesco Marincola, Kim Margolin, Candice McCoy, Graham Pawelec, John Rothman, Teresa Ramirez-Montagut, Jeffrey Schlom, Pramod Srivastava, Sarah Wallis, Steffen Walter, Ena Wang, John Waslif

**Affiliations:** 1 Kite Pharma Inc, Los Angeles, CA, USA; 2 Galena Biopharma, Inc, Lake Oswego, OR, USA; 3 Northwest Biotherapeutics, Bethesda, MD, USA; 4 Aduro Biotech, Berkeley, CA, USA; 5 Department of Medicine, Surgery and Immunology, UPCI Immunologic Monitoring and Cellular Products Laboratory, University of Pittsburgh, Pittsburgh, PA, USA; 6 California Stem Cell, Irvine, CA, USA; 7 Oxford BioMedica, Oxford, UK; 8 Norris Comprehensive Cancer Center, University of Southern California, Los Angeles, CA, USA; 9 Department of Surgical Oncology, University of California Los Angeles, Los Angeles, CA, USA; 10 Department of Transfusion Medicine; Infectious Disease and Immunogenetics Section, National Institutes of Health, Bethesda, MD, USA; 11 Division of Medical Oncology, University of Washington, Seattle, WA, USA; 12 Dendreon Corp, Seattle, WA, USA; 13 Center for Medical Research, University of Tübingen, Tübingen, Germany; 14 Advaxis Inc Genomics Institute of the Novartis Research Foundation, Princeton, NJ, USA; 15 Immunotherapeutics Group, National Institutes of Health, Bethesda, MD, USA; 16 Department of Immunology, University of Connecticut Health Center, Farmington, CT, USA; 17 Arrowhead Publishers and Conferences, Minneapolis, MN, USA; 18 Immatics Biotechnologies GmbH, Tübingen, Germany; 19 Arrowhead Publishers and Conferences, Minneapolis, MN, USA

## Abstract

While there has been significant progress in advancing novel immune therapies to the bedside, much more needs to be done to fully tap into the potential of the immune system. It has become increasingly clear that besides practical and operational challenges, the heterogeneity of cancer and the limited efficacy profile of current immunotherapy platforms are the two main hurdles. Nevertheless, the promising clinical data of several approaches point to a roadmap that carries the promise to significantly advance cancer immunotherapy. A new annual series sponsored by Arrowhead Publishers and Conferences aims at bringing together scientific and business leadership from academia and industry, to identify, share and discuss most current priorities in research and translation of novel immune interventions. This Editorial provides highlights of the first event held earlier this year and outlines the focus of the second meeting to be held in 2013 that will be dedicated to stem cells and immunotherapy.

## 

While there has been progress in translating immune interventions from the bench to the bedside – most notably monoclonal antibodies and second generation antibody drug conjugates (ADC) [1] – much more needs to be done to leverage the immune system in the fight against cancer. With the advent of checkpoint blockade antibodies such as the approval of anti-CTLA4 monoclonal antibody Yervoy™ (Ipilumumab)® [2], PD-1/PD-L blocking molecules in development [3] and the previous approval of the autologous cellular immunotherapy Provenge®, (Sipuleucel-T) [4], we are entering a new era of rapid diversification of the platform technologies that carry significant promise to change the standard of care in cancer. Key to this aspect is to identify targets and optimize approaches that mobilize the immune system safely and effectively to provide long-term control of disease in the adjuvant or post-therapy minimal residual disease, as well as in advanced, metastatic setting.

A recently published, highly accessed collaborative review [5], identified nine major hurdles in effectively designing and translating novel immune interventions for cancer, including the limited predictive value of preclinical modeling, the complexity of cancer and immune escape mechanisms reflected in the need for combination therapies, scarcity of reliable predictive and pharmacodynamics biomarkers, along with regulatory, budgetary and operational bottlenecks. Some of these scientific and technical hurdles were also discussed in more detail at a summit organized by Arrowhead Publishers and Conferences, the first in a recurring series, entitled “The World Cancer Immunotherapy Conference: Challenges and Opportunities in Clinical Development, Clinical Trial Design and Commercialization” which took place on January 25–26, 2012 in San Diego, CA (http://www.cancervaccinesconference.com/). This event brought together a focused group of key scientists and industry leadership from across the globe to share research, case studies and viewpoints on various topics integral to a better understanding of the challenges and opportunities facing developers of therapeutic cancer vaccines and immune interventions in general. The selected topics derived from five questions with a highly pragmatic connotation:

1. How can we improve the potency of immunotherapies, both from the standpoint of response rate and durability?

2. What are the feasible strategies for integrating immunotherapy with other treatments ?

3. How do we limit the high failure rate in late stage clinical development ?

4. What is the significance and value of immune monitoring ?

5. How do we identify and effectively utilize lessons learned from past challenges in clinical and commercial settings ?

Optimization of the current product development processes must benefit from prior experience especially with immunotherapies that underwent a successful cycle reaching commercialization. Dr. Candice McCoy from Dendreon Corp. outlined challenges and lessons learned from the clinical development and approval process for Provenge®. In addition to sharing clinical trial results and regulatory milestones, she discussed items of critical importance for bringing an exceedingly complex immunotherapeutic product to market: the need for immune response assessment that is relevant to the mechanism of action, and for the development of potency assay biomarkers starting early in development so that during late-stage clinical trials appropriate release testing accompanied by sound acceptance criteria can be validated, a pre-requisite for successful licensing.

Predictive biomarker discovery and translation to companion diagnostics to identify patients with a higher likelihood of benefitting from immunotherapy could make the difference between a viable and a non-viable product in both the clinic and market place. This important undertaking addresses the tremendous heterogeneity of the neoplastic molecular mechanisms, host genetic polymorphisms of the immune system and other controllers of malignancy, and demographic factors that impact the occurrence of common and rare cancers. Dr. Vincent Brichard from GSK Biologicals, while providing an update on the status of the pivotal MAGRIT and DERMA trials utilizing adjuvanted recombinant MAGE-A3 protein as a therapeutic vaccine, highlighted the potentially critical value of immune gene signatures as predictive markers and co-primary endpoints along with disease-free survival. Meeting the clinical efficacy endpoints in patient populations identified by this signature would break novel grounds in immunotherapy, and translate to market a recombinant protein based cancer vaccine accompanied by a diagnostic indicator.

Dr. Richard Harrop from Oxford BioMedica provided a retrospective analysis of the phase III trial with the TroVax® (MVA-5T-4) therapeutic vaccine in renal cancer patients. While the overall trial results did not meet the primary endpoints, they enabled a detailed investigation of early predictors of treatment benefit such as tumor antigen 5T4-specific antibodies. Markers in this category are needed for treatments that may have a delayed impact on the disease or may benefit only subsets of patients who can be defined prior to therapy. The results from the trial appeared to corroborate the value of the mean corpuscular hemoglobin concentration (MCHC), a commonly-reported clinical laboratory value, as a predictor of clinical benefit for the addition of MVA-5T4 vaccine to standard Sunitinib or cytokine therapy. This inflammatory marker and others representing the tumor, treatment, and host interactions will require validation in subsequent trials.

Dr. Steffen Walter from Immatics Biotechnologies, discussed how biomarkers have been utilized to guide clinical development of their GM-CSF-adjuvanted multi-peptide renal and colorectal cancer vaccines, including the investigation of cyclophosphamide in conjunction with vaccination, based on previous observations negatively correlating the presence and number of T regulatory (Treg) cells with the immune response upon vaccination. The much-debated and diverse topic of biomarkers was discussed in a panel session co-chaired by Drs. Walter and Francesco Marincola of the NIH, entitled “Biomarkers and Cancer Immunotherapies: Measuring success in clinical development”.

Immunologic monitoring is in itself a vast arena within the realm of biomarkers, as the immune response is a personalized and multifaceted response to the tumor as well as any intervention being tested. The complexity of this area as well as a wide range of scientific and technical approaches to it, have led to controversy as to correlations between measures of immunity and clinical outcomes. Dr. Lisa Butterfield from the University of Pittsburgh Cancer Institute, first provided an update on current immunotherapy clinical trials conducted in hepatocellular carcinoma and melanoma with a variety of vaccine approaches, based on lessons learned from previous immunologic monitoring. She then highlighted the concept that a broad and diverse immune response score encompassing the breadth of response against multiple antigens with evidence of determinant spreading, functionality and co-induction of both T helper and cytotoxic lymphocytes, could be a superior correlate of clinical activity in the face of patient and therapeutic approach heterogeneity. Dr. Graham Pawelec, from the University of Tübingen Medical School, showed a very interesting direct relationship between long term survival of melanoma patients and immunity against different tumor antigens demonstrated by functional T cell assays *in vitro*, irrespective of the patients treatment. While the CD8 T cell response against Melan A/MART-1 and NY-ESO-1 were directly correlated with survival in prospective studies, a CD4 T cell response against Melan-A/MART-1 mitigated against long-term survival, presumably due to selective activation of regulatory T cells. Dr. Francesco Marincola, in his presentation entitled “Focusing immune monitoring where it matters: the tumor site” discussed a cluster of pivotal genes expressed within the tumor and associated with tumor regression by immune mechanisms (“immunological constant of regression”). In addition, he provided evidence that IRF-1 is a key switch deploying acute rather than chronic inflammation, resulting in tumor regression rather than progression. Both Drs. Marincola and Ena Wang of the NIH discussed their tantalizing discovery that IRF-5 polymorphism is associated with clinical benefit afforded by adoptive T cell therapy, a platform technology that allows rapid progress in discovering of immune correlates of protection by virtue of its increased rate of clinical response. Illustrating the progress of evaluating various vaccine technologies in the clinical development stage, there were several presentations on topics spanning autologous cell-based, viral and peptide-based vaccines in phase II and III clinical trials. Drs. John Rothman, Advaxis Inc and Dirk Brockstedt, Aduro Biotech, highlighted the progress with their distinct Listeria vaccines aimed at treating different solid cancers. Dr. Rothman outlined the evidence to date on their HPV E7-directed vaccine for advanced cervical carcinoma, grounds for the initiation of a randomized trial in combination with platinum-based chemotherapy. Dr. Brockstedt discussed a different clinical strategy, bringing a Listeria vector attenuated by distinct genetic means and expressing the tumor antigen mesothelin to phase II trials in pancreatic carcinoma, using a heterologous prime-boost approach. Dr. Marnix Bosch, Northwest Biotherapeutics Corp., presented his company’s program advancing the DC-based platform technology primarily in advanced clinical trials in glioblastoma multiforme, with exciting new collaborative efforts exploring integration of DC vaccines with adoptive T cell therapy in ovarian carcinoma, and direct intra-tumoral injection of mature DC in head and neck cancer and other inoperable tumors. Dr. Mark Ahn, Galena Biopharma, described the progress of NeuVax™ (nelipepimut-S), a nonapeptide Her2/Neu oncogene product delivered intradermally in the minimal residual disease setting. Based on successful phase II, NeuVax is being developed in a Phase III with a Special Protocol Assessment (SPA), as an adjuvant immunotherapy to prevent or delay recurrence for women with early-stage high risk (node positive) HER2 negative (IHC 1+/2+) breast cancer.

As is now evident, vaccines are being explored more and more as combination approaches with agents that are already part of standard of care or other interventions that have immune modulating potential. This is for two reasons: to increase vaccine efficacy in the clinic and secondly, to accomplish application in earlier disease stages for both scientific and commercial reasons. Dr. Jeffrey Schlom, NIH, developed this aspect in his presentation entitled “Recombinant vector cancer vaccines as monotherapy and in combination therapies” utilizing TRICOM vaccinia vector co-expressing immune stimulatory molecules as a representative example, in context of a rapidly evolving field [6]. Dr. Kim Margolin, of the University of Washington School of Medicine, outlined the emerging collaborative efforts of “The Cancer Immunotherapy Trials Network” (CITN) to match the highest priority immune-modulating compounds with promising vaccines or immune interventions, offering a novel framework for testing combination immunotherapies.

A number of speakers discussed new and promising technologies, adjuvants and targets. Dr. W. Martin Kast of the University of Southern California presented his efforts and exciting preclinical evidence in support of heterologous prime-boost vaccines encompassing genetic and vectors expressing strong adjuvants such as LIGHT, which mobilizes immunity within tumors [7]. Dr. Tereza Ramirez-Montagut, of the Genomics Institute of the Novartis Research Foundation, introduced her organization’s efforts to develop more potent vaccine-based technologies that employ modified antigens that incorporate unnatural amino acids and novel TLR agonists. Michael Cross, of OncoSec Medical Inc., described the efforts to advance an *in vivo* electroporation device applicable to tumors that are superficial but could have metastatic manifestations. The device, in conjunction with IL-12 plasmid utilization, is entering later clinical trial stages in melanoma, cutaneous T cell lymphoma and Merckel cell carcinoma. Dr. Pramod Srivastava, of the University of Connecticut Health Center, outlined a new concept, utilization of high performance genomics to rapidly discover a personalized “immunome” – a collection of potentially immunogenic antigens and epitopes – that could be turned around rapidly into a truly personalized vaccine. The facilitating elements in support of such technologies are becoming more feasible, thus creating the premises for such a personalized approach sooner rather than later.

Tumor-initiating cells (“cancer stem cells”) are the topic of much debate and considered to represent cellular targets amenable to immune interventions. Dr. Andrew Cornforth of California Stem Cell, presented his organization’s efforts to translate a program based on rapidly proliferating tumor cells isolated *in vitro*, in conjunction with autologous dendritic cells and GM-CSF for melanoma. Dr. Jeffrey Schlom from the NIH introduced a novel target, Brachyury, involved in the epithelial-to-mesenchymal transition (EMT), cancer cell ”stemness” and drug resistance. Dr. Richard Koya of the University of California Los Angeles, discussed a very exciting emerging approach, utilizing genetic engineering and adoptive transfer of T cells for treatment of solid tumors such as melanoma. Promising clinical data, hurdles and opportunities to integrate this approach with other therapies, especially novel FDA-approved inhibitors of the MAPK signaling to achieve durable management of cancer, were also discussed.

Last but not least – and reflecting the pragmatic focus of the summit – Mara Goldstein, Senior Healthcare Analyst with Cantor Fitzgerald, provided “An Investor's Perspective on Cancer Immunotherapy Research & Development”. Her discussion highlighted that investors are gradually becoming more optimistic, reflected by an environment in which initial public offerings and financing in general are possible and even oversubscribed, yet often discounted.

As is apparent from the above report, the field of cancer immunotherapy is rapidly expanding and diversifying; thus Arrowhead Publishers and Conferences is now organizing the 2nd Annual Arrowhead Cancer Immunotherapy Conference that will occur on April 4-5th, 2013, in the Washington DC area (http://www.cancervaccinesconference.com/). More details of this event will be released shortly, but the overall theme will be “Stem Cells and Cancer Immunotherapy”. The first track will cover tumor initiating or “cancer stem cells” as a new category of targets for immune intervention and drug development. Recent breakthrough evidence is in strong support of the concept of cell “stemness” in solid tumors [8-10] and stem cell-directed immunotherapy as an alternate and potentially more effective approach to tackle cancer [11]. In this track, speakers will be covering biology and identification of cancer stem cells or tumor initiating cells, and associated targets amenable to a variety of platforms including antibodies, vaccines or cells.

The second track will be dedicated to lymphoid and hematopoietic stem cells that have demonstrated a therapeutic potential in the context of genetic engineering of the immune system and adoptive T cell therapy [12,13]. This approach carries the considerable potential of effectively halting the progression of cancer in its latest stage, and the most important tasks ahead are how to control its safety and efficacy, along with increasing its feasibility. The key ingredients for the next stage in development of this approach are becoming more available as the realization that a new artificial immune repertoire – with reactivity against cancer antigens - could be engrafted by genetic means and stem cells onto the natural immune repertoire that evolved primarily as an anti-infectious rather than anti-cancer defense mechanism. The practical question of how to effectively harness renewable army of anti-tumor effector cells, at the time and place needed, awaits appropriate solutions as this will be crucial for safety and efficacy. Thus, in this track, speakers will be covering aspects related to the translation of adoptive T cell therapies and strategies to redesign the immune system through genetically-engineered hematopoietic stem cells or “stem cell-like memory cells”.

## Competing interest

AB declares no competing interests. The authors have academic or industrial affiliation as disclosed.

## Authors’ contributions

AB conceived the manuscript and all co-authors provided necessary input. SW and JW organized the meeting on behalf of the Arrowhead Publishers and Conferences. All authors read and approved the final manuscript.
